# Can a Sediment Core Reveal the Plastic Age? Microplastic Preservation in a Coastal Sedimentary Record

**DOI:** 10.1021/acs.est.2c04264

**Published:** 2022-11-14

**Authors:** Laura Simon-Sánchez, Michaël Grelaud, Claudia Lorenz, Jordi Garcia-Orellana, Alvise Vianello, Fan Liu, Jes Vollertsen, Patrizia Ziveri

**Affiliations:** † Institute of Environmental Science and Technology (ICTA), Autonomous University of Barcelona (UAB), Bellaterra08193, Spain; ‡ Department of the Built Environment, Aalborg University, Thomas Manns Vej 23, Aalborg Øst9220, Denmark; § Departament de Física, Universitat Autònoma de Barcelona, Autonomous University of Barcelona (UAB), Bellaterra08193, Spain; ∥ Catalan Institution for Research and Advanced Studies (ICREA), Pg. Lluís Companys 23, Barcelona08010, Spain

**Keywords:** microplastics, sediments, weathering, carbonyl index, accumulation

## Abstract

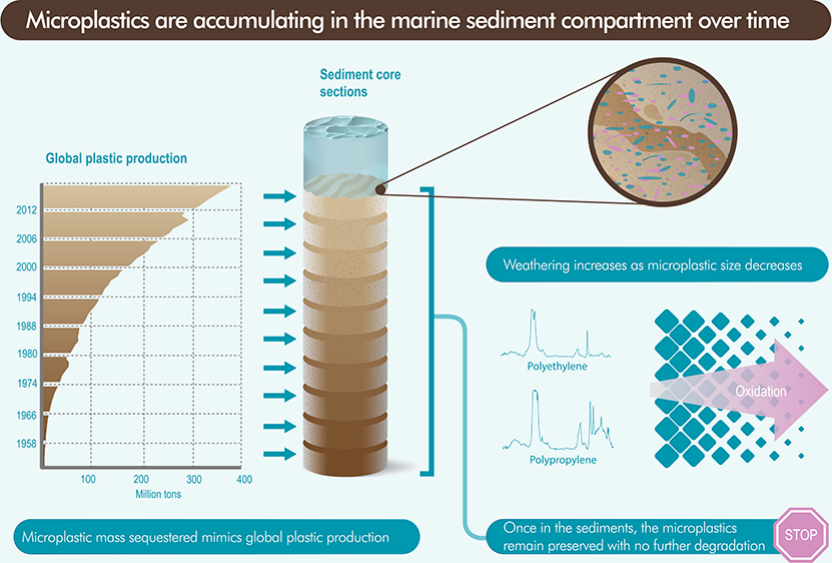

The seafloor is the major sink for microplastic (MP) pollutants. However, there is a lack of robust data on the historical evolution of MP pollution in the sediment compartment, particularly the sequestration and burial rate of small MPs. By combining a palaeoceanographic approach and state-of-the-art analytical methods for MP identification down to 11 μm in size, we present the first high-resolution reconstruction of MP pollution from an undisturbed sediment core collected in the NW Mediterranean Sea. Furthermore, we investigate the fate of MPs once buried in the sediments by evaluating the changes in the size distribution of the MPs and the weathering status of the polyolefins, polyethylene, and polypropylene. Our results indicate that the MP mass sequestered in the sediment compartment mimics the global plastic production from 1965 to 2016. We observed an increase in the weathering status of the polyolefins as the size decreased. However, the variability in the size and weathering status of the MPs throughout the sedimentary record indicated that these pollutants, once incorporated into sediments, remain preserved with no further degradation under conditions lacking remobilization.

## Abstract 2

Microplastics sequestered into undisturbed marine sediments remain preserved with no signal of further degradation over time.

## 1 Introduction

The proposed Anthropocene epoch frames the geological time when intensified anthropogenic activities induced mounting changes in Earth-system processes.^1^ This period has been characterized by manufacturing new materials that are indispensable in our societies. Among these materials, plastics are unique. Not only does the global plastic mass outstrip the living animal biomass on Earth,^2^ but the rate of production and disposal of these materials already exceeds the planetary boundary.^3^ Moreover, the durability of plastics favors their preservation as potential long-lasting distinctive markers in sedimentary records.^4^ The increasing presence of plastics in our oceans raises concerns about the harm they represent to the functioning of ecosystems, from alterations to the marine carbon cycle^5^ to individual ecotoxicological damage.^6^ Nonetheless, elucidating the dispersion and accumulation of microplastics (MPs; 1–5000 μm^7^) and the elusive nanoplastics (NPs; <1 μm) remain challenging as fluxes, fate, and residence time are still poorly understood, partly due to the constraints of available analytical methods.^8,9^ The growing body of data on the presence of MPs in the marine environment points to the seabed as a significant sink for these pollutants.^10,11^ The mechanisms for reaching this deep environment are primarily related to the density of the MPs, as buoyant MPs are expected to float at sea. However, MPs can sink in the water column^12^ and be transported by deeper currents.^13^ Furthermore, physicochemical changes caused by fragmentation, weathering, and biological interaction, such as ingestion–egestion, aggregation with organic and inorganic matter, and biofilm formation, might facilitate the export of MPs to the ocean floor.^14^ Once on the seafloor, the fate, depositional trends, and environmental degradation of MPs, especially the smaller fraction (<300 μm), remain undetermined.^15^

MPs buried in sediments can interact with benthic biota.^16,17^ These pollutants potentially can also be used as a chronological tracer of sedimentary records.^18^ As such, MPs may be effective as a temporal tracer; however, their preservation and degradation in sediments have not been explored. Depositional environments, such as river prodeltas, offer high-resolution stratigraphy over recent decades, making them suitable to track the evolution of MP pollution. Moreover, rivers play an essential role as a source of MPs to the open sea and hold a relevant storage capacity for these pollutants.^19^ In this study, we investigate MP accumulation over time as well as the fate of these pollutants once buried in sediments. We combined different analytical approaches to reconstruct the accumulation of small MPs (11–1000 μm), including sedimentary geochronological methods and state-of-the-art FPA-μFTIR-Imaging (Focal Plane Array-Fourier Transform Infrared-Imaging-Micro-Spectroscopy). The radiometric analysis (^210^Pb,^137^Cs) provides robust chronologies for the Plastic Age (i.e., post-1950s) with years to decades resolution,^18^ while FPA-μFTIR-Imaging spectroscopy allows reliable detection of particles down to 11 μm in size, avoiding analyst’s bias.^20^ In addition, we explore the degradation status of these pollutants once buried in the continental shelf by quantifying the weathered status of the polyolefins, polyethylene (PE), and polypropylene (PP). The investigated polymers represented, as of 2015, 50.3% of the global plastic waste generation,^21^ and despite their positive buoyancy, PE and PP are the most abundant polymers reported in sedimentary environments.^22,23^ We discuss our findings in the context of previous MP observations, the variability of MP properties over time, and the environmental factors leading to the sequestration and preservation of MPs in the seabed.

## 2 Methods

### 2.1 Core Material

**Figure 1:**
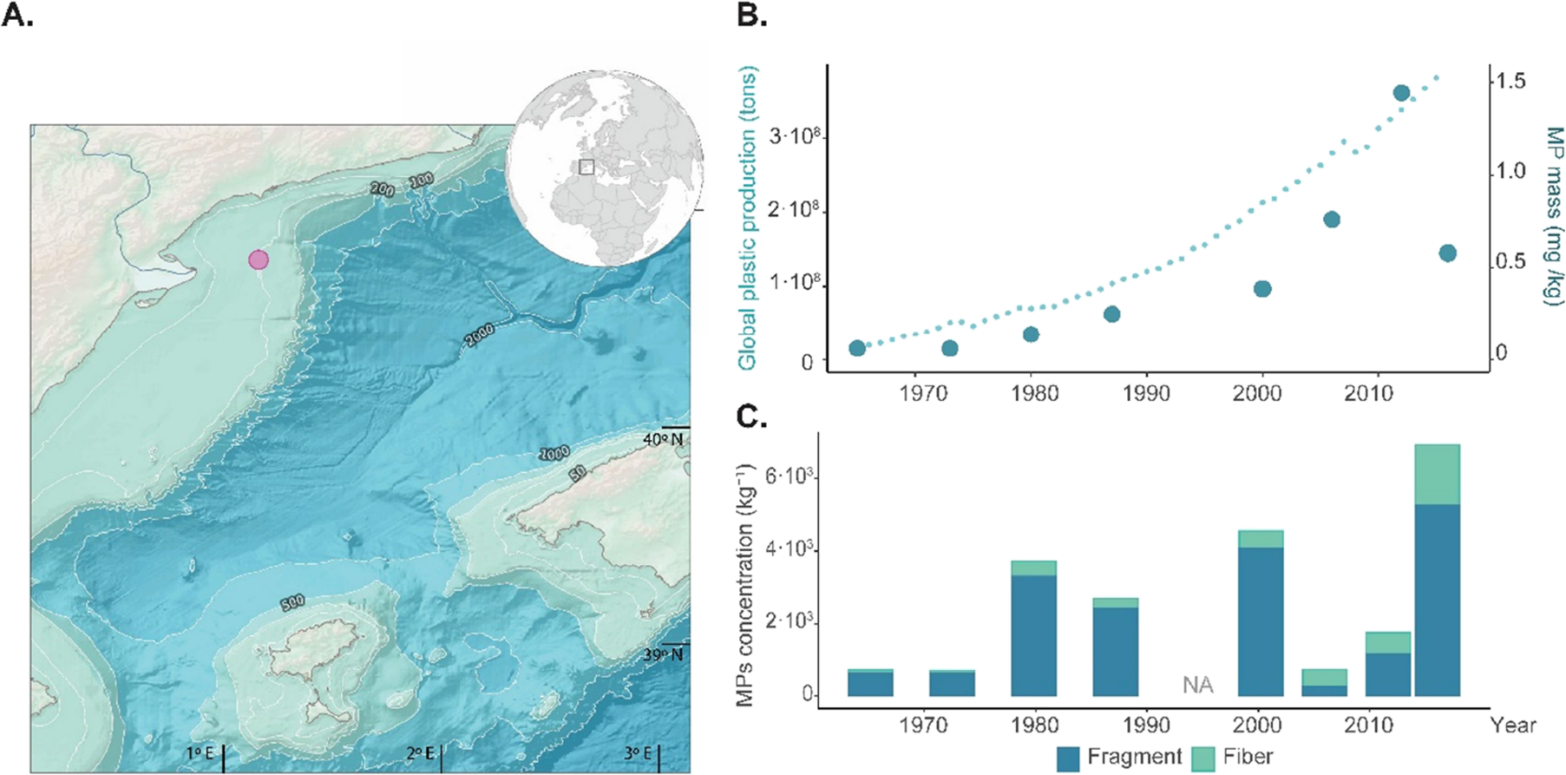
(A) Geographical location of the sampling station. (B) Abundance of mass-microplastic registered in the sediment core (*y*-axis right, units MP mass: mg kg^–1^), against the global plastic production (*y*-axis left) from 1965 to 2016. (C) Abundance of microplastic (number) classified as fragments and fibers, from 1965 to 2016.

A total of five sediment cores (K/C Denmark Multi Corer) were extracted in the Balearic Sea (NW Mediterranean) during the MERS_BI cruise in November 2019 onboard the R/V Sarmiento de Gamboa. The cores were age-profiled as described below and the core showing the most constant and continuous sediment accumulation rate was chosen for further analysis, assuming that this core was the one least disturbed by bioturbation and seismic activities. The selected sediment core ST17_MUC2 (40.7726°N, 1.1643°E; 104 m water depth; 37 cm in length; Figure 1A) was sectioned into 1 cm intervals. The samples were transferred to tared low-density polyethylene (LDPE) zip lock bags and stored at 4 °C. In the laboratory, each section was homogenized and dried at 50 °C until a constant Dry Weight (DW) was reached. An aliquot (3–5 g) was separated from each section for geochronological analysis.

### 2.2 Age Model for ST17_MUC2

Dried sediment samples were analyzed to determine ^210^Pb specific activities by α-spectrometry through the analysis of its granddaughter ^210^Po at the Grup de Recerca en Radioactivitat Ambiental de Barcelona (GRAB) at the Universitat Autònoma de Barcelona—following the method described in ref (24). After adding ^209^Po as an internal tracer, 200–300 mg of sediment aliquots were totally digested in acid media using an analytical microwave oven, and Po isotopes were plated on silver discs in HCl 1 N at 70 °C while stirring for 8 h. Alpha emissions of ^210^Po and ^209^Po were measured using Passivated Implanted Planar Silicon detectors (PIPS; CANBERRA, Mod. PD-450.18 A.M.).

The age model derived from ^210^Pb was obtained using the Constant Supply: Constant Flux (CS:CF) model.^25^ The mean mass accumulation rate (MAR; g cm^–2^ year^–1^) was obtained by performing a non-linear least-square fit between the excess ^210^Pb (Bq kg^–1^) and the cumulative dry mass (g cm^–2^) of the sediment core. The excess ^210^Pb (^210^Pb_xs_) was determined by subtracting the constant ^210^Pb supported, which was estimated as the average ^210^Pb concentration of the deeper sediment layers analyzed, wherein ^210^Pb activities reached constant values. To calculate the sedimentation rate (SR; cm year^–1^), the MAR was divided by the dry bulk density (DBD) of the section, where the DBD of the section is obtained by dividing the DW mass (g) of the slice by its volume (cm^3^).

The activities of ^137^Cs were measured using an HPGe **γ**-spectroscopy detector (CANBERRA, Mod. GCW3523 ad Mod.SAGe Well). Aliquots of dry sediments (1–3 g) were placed into PE counting vials using calibrated geometries and counted for around 186,000 s.

### 2.3 Microplastic Analysis

The upper 10 cm of the sediment core, corresponding to deposits since the beginning of the plastic era, was cut up at 1 cm resolution and analyzed for MP content following the protocol described in Liu et al.^26^ and the Supplementary Material. Unfortunately, the first section (9–10 cm) was lost in the sample preparation, and therefore the data present 1965 and onward. The sediment mass processed for MP analysis varied between slices, ranging between 22.8 and 59.5 g (Table S1). Briefly, the sample matrix was removed using a multi-step sample treatment, including pre-oxidation, density separation, buffered multi-enzymatic treatment, and catalyzed oxidation. The resulting isolated particles were transferred into a glass 10 mL headspace vial with HPLC grade 50% ethanol, and the solvent was evaporated using an evaporator (TurboVap LV, Biotage). Finally, a fixed volume (3 mL of HPLC grade 50% ethanol) was used to re-mobilize the particles. To minimize data extrapolation, the total volume of each sample was deposited onto multiple windows (four to five per sample) and analyzed. For that, the sample was homogenized (vortex), and multiple aliquots of the suspension were deposited with a capillary glass pipette (micro-classic, Brand GmbH, Germany) onto an area of 78.5 mm^2^ (⌀10 mm) of a zinc selenide window (ZnSe—⌀13 mm, 2 mm thickness, Crystran LTD, UK) held by a compression cell (Pike Technologies, USA). The deposited sample was dried overnight at 55 °C prior to analysis.

Sample analysis was carried out using FPA-μFTIR-Imaging spectroscopy. Measurements were performed using an Agilent 620 FTIR microscope equipped with a 128 × 128 pixel MCT-FPA detector (Mercury Cadmium Telluride—Focal Plane Array) coupled with a Cary 670 FTIR spectrometer (Agilent Technologies, Santa Clara, CA, USA). Optical images of the ZnSe window were determined with a 15× objective. The IR map was collected in transmission mode in the range of 3750 to 850 cm^–1^, using a 15× IR Cassegrain objective-condenser system with a spectral resolution of 8 cm^–1^, 30 co-added scans for the sample, and 120 for the background. This setup allowed measuring the whole area of the ZnSe window (⌀10 mm, 78.5 mm^2^) with a pixel resolution of 5.5 × 5.5 μm.

The resulting hyperspectral images were analyzed for systematic automated MP identification with the software siMPle.^27^ After converting the recorded spectra from %Transmittance (%T) to Absorbance (Abs) and performing baseline correction,^28^ the software chemically identifies the particles on the sample by comparing every pixel of the IR map with a custom-built library containing 441 spectra of inorganic and organic materials. The resulting scores are then used to provide a material-based map of the sample, quantitative data on particle abundance, and detailed physicochemical information for each particle (polymer composition, two-dimensional size, estimated volume, and mass). The mass of an MP is estimated by equivalenting the MP by an ellipsoid and applying the density of the determined polymer type as described in references (29, 30).

### 2.4 Contamination

The sample preparation was conducted inside a laminar flow bench (Telstar AV-100), and cotton lab coats were worn. All the reagents (e.g., H_2_O_2_, ZnCl_2_, NaOH, FeSO_4_, and enzymatic buffers) were filtered through a glass fiber filter (0.7 μm, Whatman) prior to use. Only glassware or stainless-steel materials were used whenever possible, except for the density separation step, where silicon tubes and polytetrafluoroethylene (PTFE) stopcocks were unavoidable. All the materials were carefully rinsed three times with MilliQ and immediately covered. In addition to these measures, one analytical blank was run alongside each set of samples to assess contamination.

A section of the core below the Plastic Age (pre-1950s, 34–35 cm) was selected and analyzed along with the samples to account for potential contamination during sampling. We assumed that the MP recovered in this section represents the potential contamination during the coring and storing as the existence of plastic materials in these sections would correspond to the 19th century, according to the age model.

### 2.5 Data Analysis

For each sample, the results of each ZnSe window were combined and corrected for contamination. The contamination recorded for the sampling and in the analytical blanks were subtracted from the samples, considering the size class and polymer composition. The total amount of MPs (items kg^–1^ DW and mg kg^–1^ DW), MP fluxes (items m^–2^ year^–1^ and mg m^–2^ year^–1^), and polymer diversity was then calculated. The shape of particles was classified as fragments or fibers, according to Vianello et al.,^31^ while size classes were adopted from Lorenz et al.^32^ MP burial rates (items m^–2^ year^–1^) were calculated by multiplying the MP concentration by the MAR of the core and standardized to m^2^. Polymer diversity across the sediment core was assessed using the Shannon–Wiener diversity index (*H*′) and Pielou’s evenness index (*J*′). The data normality of the dataset was tested using the Shapiro–Wilk test. Non-parametric tests (Kruskal–Wallis, followed by a post hoc Wilcoxon–Mann–Whitney) were applied to reveal differences. Data analysis and figures were produced in QGIS Desktop 3.12 ’Bucureşti’^33^ and R-4.1.1,^34^ using ggplot^35^ and cowplot^36^ packages. The level of statistical significance was set at *p* < 0.05.

### 2.6 Carbonyl Index

The degradation status of polyolefins (PE, PP) was investigated using the carbonyl index (CI), which allows the identification of the chemical changes in several polymeric materials by targeting the specific absorption band of the carbonyl species produced mainly during thermo- and photo-oxidation.^37,38^ The CI was computed adopting the Specified Area Under Band (SUAB) methodology,^39^ applying the equation:
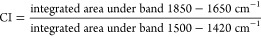
1

The spectra of the particles were recorded in transmission mode through the entire particle’s thickness, including the spectral contribution from the particle’s inner core. All the spectra belonging to PE and PP particles were automatically exported from each sample dataset using a custom-design feature in siMPle. The exported spectra were loaded into SpectraGryph 1.2.15 software;^40^ the integrated areas under the selected bands were calculated using the peak analysis tool and used to compute the CI measurements.

## 3 Results and Discussion

### 3.1 Sediment Archive

The ^210^Pb_xs_ profile of the sediment core showed an almost constant and continuous sediment accumulation rate, indicating an undisturbed condition of the core (Figure S1). The application of the CF–CS dating model resulted in an SR of 0.12 ± 0.01 cm year^–1^. The age model determined the beginning of the Plastic Age (50s) around 10–11 cm (1951 ± 3 year). The artificial radionuclide ^137^Cs used to validate the geochronology did not provide discernible activities. This limitation can be related to the limited sediment mass analyzed, the decay of ^137^Cs, the low concentration in sediments, and its potential mobility.^41^ Nevertheless, in its absence, historical events can corroborate the ^210^Pb geochronology. The Ebro River discharge records showed two marked trends after 1951, and the maximum recorded in 1959, which were followed by a significant decrease in the annual contribution within the following years (Figure S2). Remarkably, the Ribaroja and Mequinenza dams located around 100 km upstream, whose constructions dated from 1958 to 1966, heavily impacted the Ebro River’s water and sediment flow balance.^42^ The anomaly of ^210^Pb_xs_ observed in the general exponential trend between 8.5 and 10.5 cm corresponds to the years 1965 ± 2 and 1951 ± 3, coinciding with the years of construction of the dams, a fact that adds confidence to the chronological model obtained.

### 3.2 Microplastic Concentration

MPs were successfully extracted and analyzed in 9 out of the 11 samples, except for two of them (4–5 cm and 9–10 cm) that were lost during the sample preparation. MPs were found in all the investigated sections of the sediment core and control samples. The procedural blanks contained 7 and 13 MPs, revealing polyester (PET), PP, and polystyrene (PS) contamination. In the pre-Plastic Age section (34–35 cm), seven MPs were recovered, showing contamination by PET, PE, and PP (Table S1). A total of 902 MPs (1.39 × 10^2^ μg) were recovered, considering the blank corrections (Table S2). Figure 1 shows the MP concentration in the sediment core recovered from the Ebro prodelta. The total MP abundance ranged from 706 to 6939 items kg^–1^ DW. The mass concentration ranged from 0.05 to 1.43 mg kg^–1^ DW. The highest and lowest concentrations were found at the surface of the core (0–1 cm) and in section 7–8 cm, respectively. The highest mass concentration was recorded in section 1–2 cm of the core, whereas the lowest was in the 8–9 cm.

To our knowledge, there are no previous observations in the literature of MP abundance in sedimentary records combining palaeoecological approaches (i.e., radiometric analysis, varve counting) with measurements of imaging μFTIR for MP identification. However, this method has previously been applied to characterize the MP presence in marine sediment samples, where the upper 5 cm of the seabed were retrieved using sediment corers^22,43^ or a Van-Veen grab sampler.^32^ We calculated the MP abundance for the top 4 cm (3122 items kg^–1^) of our sediment core for comparison. In the remoteness of the Kamchatka trench, northwest Pacific Ocean, MP concentrations were one to two orders of magnitude lower (14–209 items kg^–1^)^22^ than our findings. Similarly, lower concentrations (3–1189 items kg^–1^) were reported in the southern part of the North Sea.^32^ In contrast, higher MP concentrations (42–6595 items kg^–1^) were reported at the Fram Strait, west of Svalbard.^43^ In the Mediterranean Sea, the closer analytical methods can be attributed to Vianello et al.,^44^ who found relatively lower values (672–2175 items kg^–1^) in the sediments of the Lagoon of Venice, Italy. These observations agree with our previous results^19^ that despite the Ebro River being a critical system for understanding the MP fluxes entering the Mediterranean Sea and being under the influence of the Gulf of Lion current, the MP pollution levels in this system are intermediate to low.

### 3.3 Microplastic Sequestration and Burial Rate

The accumulated MP inventory since 1965 ± 2 was 1.44 × 10^6^ items m^–3^ (0.22 g m^–3^). MP burial rate ranged from 865 m^–2^ year^–1^ in 1973 ± 2 to 8507 m^–2^ year^–1^ in 2016 ± 1. The MP burial rate has increased by 973% since 1965, with an average standardized rate of 18% per year. The mass of MPs sequestered in the sediments ranged from 0.061 mg m^–2^ year^–1^ in 1965 ± 2 to 1.76 mg m^–2^ year^–1^ in 2012 ± 1. Kaandorp et al.^45^ calculated the sinking flux of plastics, considering all size classes, in the Mediterranean Sea from 2006 to 2016. They estimated that the sinking plastic fluxes from the Algerian to the Spanish coast ranged from 0.1 to 1.0 g km^–2^ day^–1^. Our results showed that in 2016 ± 1, the sinking mass of small MPs almost doubled their higher estimated value (1.89 g km^–2^ day^–1^). The relevance of the study area should be noted as river deltas are vulnerable systems subjected to upstream anthropogenic stressors and are recognized accumulation areas for several pollutants.^19,46^

### 3.4 Relevance of Mass Unit on Microplastic Sequestration

In contrast to previous studies,^15^ no significant correlation was found between the abundance of MPs (items kg^–1^) and the sediment depth (Pearson’s, *r* = −0.52, *p* = 0.19; Figure 1C). Formerly, the MP fluxes reaching and accumulating in the sediment compartment have been reported to directly correlate with global plastic production, growing population,^47^ and landscape changes by using plastic materials (e.g., greenhouses^48^). Reconstructing the MP export (number of particles) to the benthic environment based on the plastic production and waste generation requires the assumption that MP deposition is spatially homogeneous, constant, and increasing exponentially over time. In general, MP studies reported patchiness in the spatial occurrence of these pollutants across different environmental compartments.^49−51^ When comparing the MP abundances at the sea surface and the sediments lying beneath, the concentrations and polymer composition significantly differed.^32^ The assumption of constant deposition of MPs oversimplifies the complex and diverse compounds that MPs are. MPs comprise a wide size range (1–5000 μm), morphologies, specific densities, and multifaceted chemical compositions^7,52^ that undoubtedly affect their behavior and fate under natural environmental conditions.^53^ Despite an incontestable increasing trend, the MP sequestration (number of particles) of our study showed intra-variability over the last 54 years (Figure 1C). This variability was likely driven by the synergistic combination of the heterogeneous distribution of MPs along the surface waters of the Mediterranean Sea^54^ and the different mechanisms, which are still poorly understood, leading the MP export from the surface to the benthic environment^14^ (e.g., marine snow, biofilm formation, aggregates, ingestion–egestion, vertical migration of species, deep-ocean currents, ocean turbulence). In our sediment record, the median MP size (58.7 μm, *Q*_1_–*Q*_3_: 41–91 μm) agrees with small MPs’ prevalent dominance in the sediments.^55^ The evidence of small MPs in the surface water compartment is scarce, which may be explained (i) by the constraints on the current predominant sampling methods (i.e., Neuston nets) and (ii) by the vertical transport of MPs. In the literature, there has been reported a mismatch in the size-abundance distribution of MPs floating in the surface waters (empirical data) and the expected concentrations derived from fragmentation models, showing a dearth of small MPs in this compartment.^56,57^ Emerging studies investigating the MP occurrence along the water column showed a higher relative abundance of small MPs as depth increased,^58−60^ highlighting the need for further MP knowledge along this vast compartment of the ocean to identify processes leading the vertical transport and to understand if these mechanisms are polymer- and size-selective by which the MP composition sequestered in the sediments may be influenced. Nonetheless, to compare and assess the plastic sinking fluxes and sequestration in the sediments regarding the global mass plastic produced, the unit MP mass concentration is more appropriate than the number of particles. The estimated MP mass sequestered in our sediment core over time showed a significant trend with sediment depth (Pearson’s, *r* = −0.79, *p* < 0.05), as well a similar exponential trend was observed between the MP mass sequestered in the sedimentary records of Ebro prodelta and the global mass plastic production (Figure 1B).

### 3.5 Microplastic Characterization

Most MPs found in the sediment core were fragments (Figure 1C), accounting for 83.3% of the total particles. Similar findings were reported in sediment cores collected in the North and Celtic Sea, where the MP identification was done by the Nile red method.^61^ In contrast, fibers were exclusively found in the sediment records recovered in the Donghu urban lake, China.^62^ Similarly, fibers were the predominant MP morphology in the sedimentary records investigated in the Santa Barbara Basin^47^ (77.0%) and in the Rockall Trough,^63^ North Atlantic Ocean (89.0%). Noteworthy, the methods described in these studies involved visual presorting and isolation of the potential plastic particles for spectroscopy analysis. This visual approach is prone to analyst bias as fibers are easier to recognize than smaller MP fragments.^64^ Moreover, fibers are one of the primary sources of contamination in MP analysis.^65^ Thus, robust and quantitative protocols are essential to prevent contamination during sampling and sample preparation.

**Figure 2:**
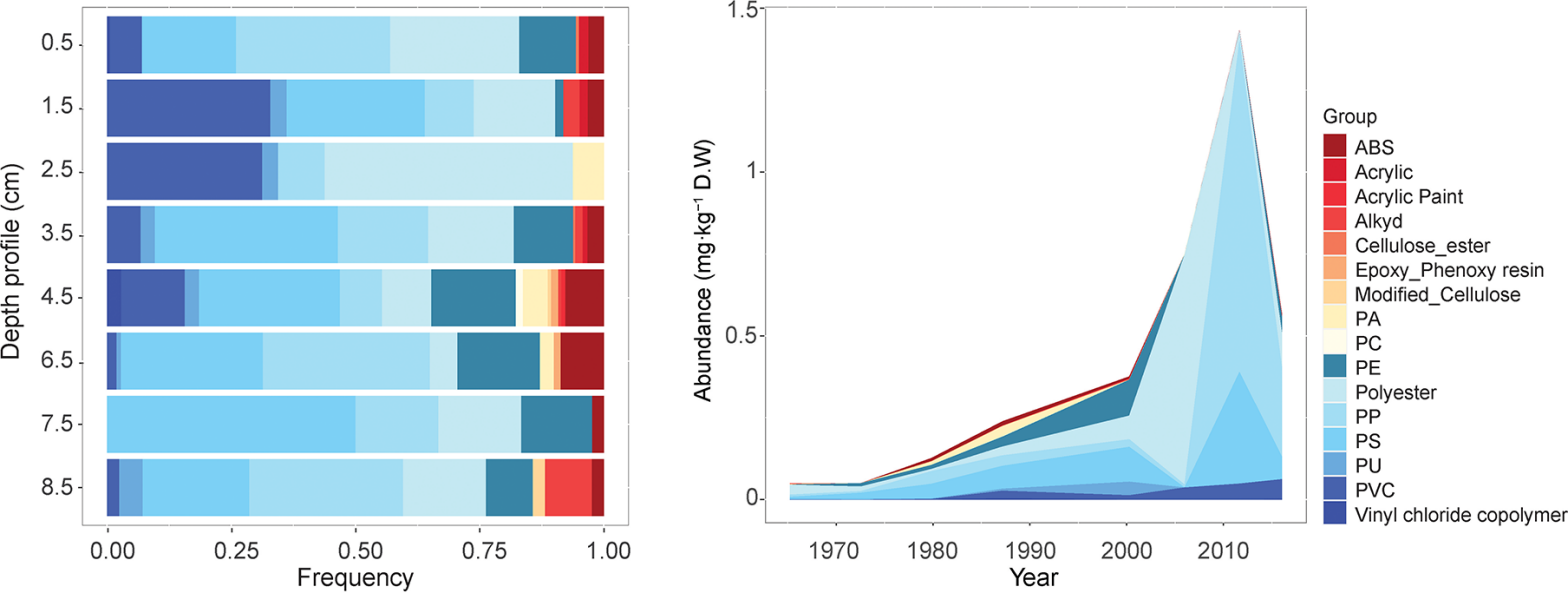
(A) Microplastic polymer composition in the sediment profile. (B) MP mass (mg kg^–1^) in the sediment core according to polymer composition from 1965 to 2016.

A total of 16 different synthetic polymers were identified in the sediment core (Figure 2). The most abundant were PS (28.4%), followed by PP (22.4%), PET (15.9%), PE (12.6%), polyvinyl chloride (PVC; 8.5%), acrylonitrile butadiene styrene (ABS; 5.9%); polyurethane (PU; 1.9%), polyamide (PA; 1.7%), alkyds (1.1%), and others (2.5%). The number of different synthetic polymers in each section ranged between 5 and 14 (Figure 2A). The Shannon–Wiener (*H*′) values ranged from 1.0 in section 7–8 cm to 2.1 in section 5–6 cm, corresponding to 1973 ± 2 and 1987 ± 1, respectively. No significant trend was found between the polymer diversity and sediment depth (Pearson’s, *r* = −0.04, *p* = 0.912). The synthetic polymer diversity evenness (*J*’) was equal (*J*’ = 0.8) in all the sections investigated, except for section 7–8 cm (*J*’ = 0.6). Notably, the MP mass sequestered in the sediments sorted by the polymer (Figure 2 B) showed a similar exponential growth by the polymer group until early 2000. After 2006 ± 1, the major mass contribution is driven by the sequestration of PP (43.6%), PET (35.8%), and PS (14.0%).

### 3.6 Carbonyl Index

**Figure 3:**
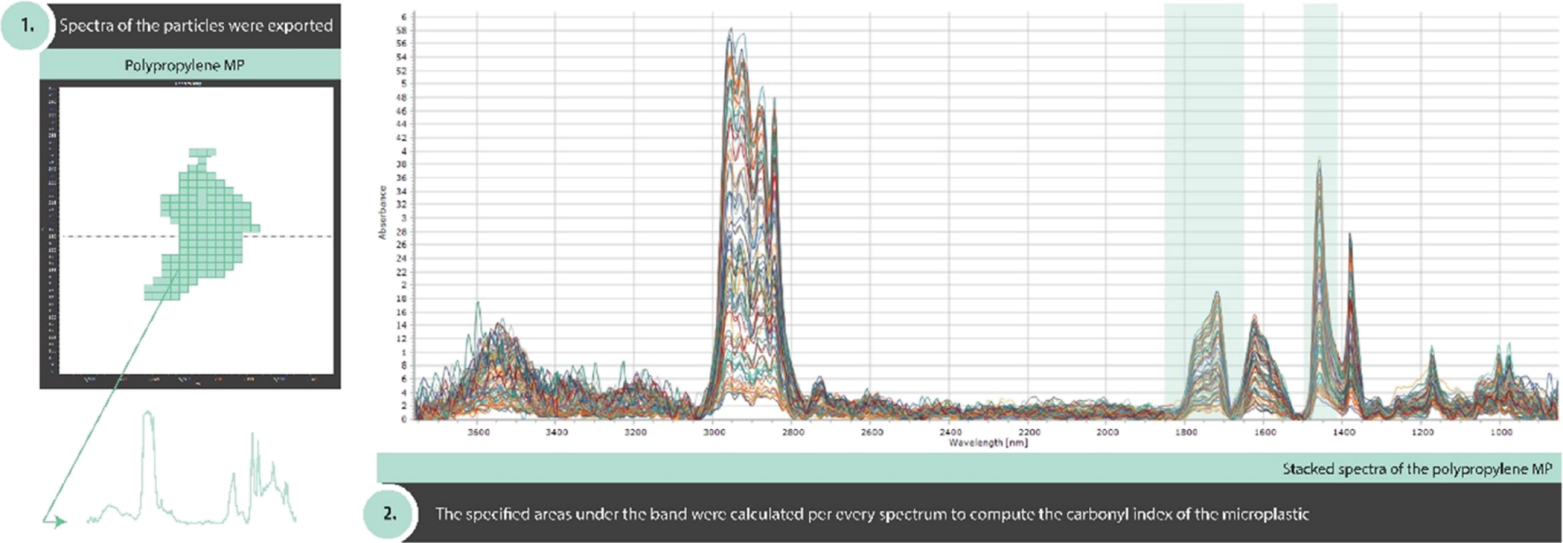
Illustrative example of the calculation of the carbonyl index for a polypropylene particle. From left to right, the image on the visualization of a particle, every pixel represents a collected spectrum. Spectra were exported from the siMPle software and treated in SpectraGryph 1.2.15 where the area below the band was computed.

FTIR spectroscopy is one of the most common techniques for MP characterization.^9^ The potential of this technique was broadened to assess the aging of the polymers under natural environmental conditions^38,66^ and accelerated weathering in the laboratory.^67^ The measurements are generally gathered with attenuated total reflection (ATR)-FTIR recording the changes occurring at a single point of the particle’s surface (limited to particles >300 μm in size). Notably, the degradation of the particle may not occur evenly across the MP. This study collected spectra of small MPs (11–1000 μm) in the transmission mode. Under this setup, the limitation of the measurement is defined by the thickness of the particle as the IR light passes through the sample, recording the particle’s inner core as well as its surface. However, by using hyperspectral images, where several spectra are measured per particle (Figure 3), it is possible to characterize the weathering status across the whole dimension of an MP in contrast to ATR-FTIR measurement. Furthermore, the method shows the potential of transmission measurements for computing the CI of small MPs in a standardized and automatic manner. A total of 246 polyolefin particles were used to compute the CI, 83 PE, and 172 PP particles. These particles yielded a total of 11,337 and 21,712 spectra, respectively. Due to non-normal distribution, the median value was computed to describe the CI per particle. The overall median CI for PE was 1.05 (*Q*_1_–*Q*_3_: 0.90–1.25) and for PP was 0.48 (*Q*_1_–*Q*_3_: 0.23–0.83). The SUAB method produces significantly higher CI values than previous methods, which statistically rejects the results’ intercomparison.^39^ No other study has previously used this method to investigate MP’s weathering under natural environmental conditions. Potrykus et al.^68^ applied it to characterize the chemical modifications on a PP plastic sample after five years of degradation in a landfill. The authors reported a CI ranging from 0.37 to 1.29. However, it should be noted that plastic degradation in seawater occurs much slower than when exposed to sunlight in dry conditions.^37^

**Figure 4:**
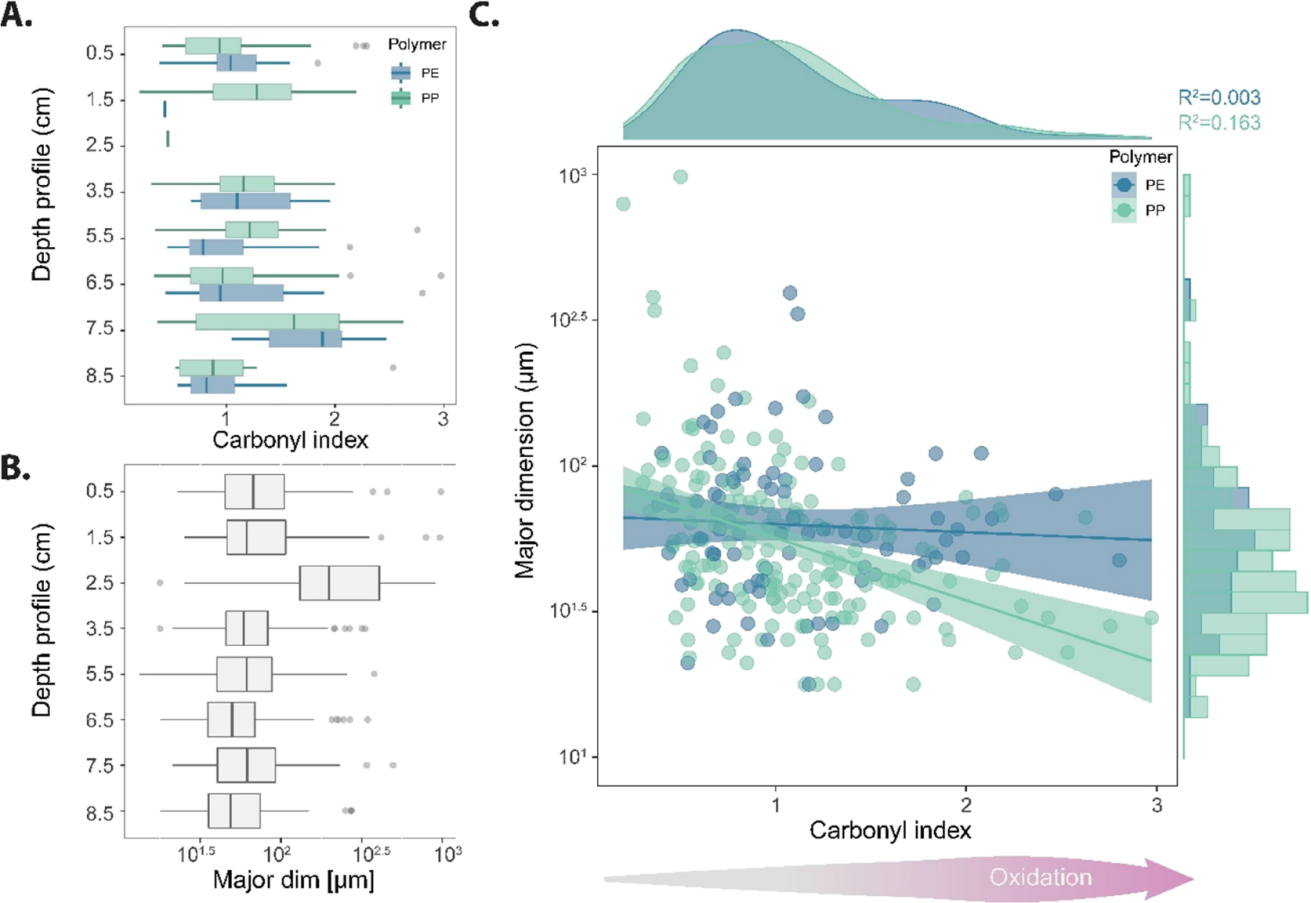
Summary of the MP weathering status and size variability in the sedimentary record A. Boxplot of the carbonyl index in the sediment profile classified as polyethylene (dark blue) and polypropylene (light blue). (B) Boxplot of the size variability (*y*-axis logarithmic scale) in the sediment core. C. Increase of the oxidation status of the polymers, measured as carbonyl index, as the particle size decreases (*y*-axis logarithmic scale).

Along with the depth profile (Figure 4A), no significant differences were found for PP (Kruskal–Wallis, chi-squared = 11.379, df = 6, *p* = 0.077). In contrast, significant differences were found for the CI of PE (Kruskal–Wallis, chi-squared = 15.732, df = 6, *p* < 0.05), between sections 5–6 cm and 7–8 cm (*p* < 0.05). Several uncertainties limit the explanation of the CI variability over time. First, although MP oxidation may occur in benthic environments under aerobic conditions, the oxygen-rich products that allow CI estimation mainly occur under light-exposure conditions. Furthermore, this process is severely retarded in seawater and further impaired by biofilm formation.^37^ Second, the tendency for polyolefins to be affected by photooxidation is defined by the original chemical composition that might vary highly depending on the additives (plasticizers, retardants, antioxidants, stabilizers) used during their manufacturing. In this context, the interpretation of CI results is dependent on the uncertainties of the initial chemical composition, the distance to the sources, and the exposure time to natural conditions before its sequestration into the sediments.

Linear regression using the CI of PE and PP, and the major dimension of these polyolefins (logarithmically transformed) indicated a tendency that smaller MPs were more oxidized (Figure 4C). However, no significant correlation was found (PE: *R*^2^ = 0.003 and PP: *R*^2^ = 0.160). Overall, this observation agrees with the conclusion that MP degradation leads to the embrittlement of a particle, which favors the fragmentation of these pollutants.^37^ Our results indicate that this process occurs prior to sequestration into the sedimentary compartment, where MPs are accumulating with no signal of further physical degradation.

### 3.7 Is MP Degradation an Active Process in the Sediment Compartment?

In our sediment core, the MP size, measured as the particle’s major dimension, presented a non-normal distribution (Shapiro–Wilk normality test, *W* = 0.52413, *p* < 0.001), ranging from 13 to 983 μm. Other MP measures, such as the area the particle took up in its imaged two-dimensional projection, could have been used to characterize MP size. We chose the major dimension (its length) to follow the criteria suggested by Hartman et al.^52^Figure 4 displays the MP size along with the sediment core. Significant differences in the MP size were found between the sections investigated (Kruskal–Wallis, chi-squared = 76.54, df = 7, *p* < 0.001). The subsequent post hoc Wilcoxon–Mann–Whitney test revealed that the MP size in section 2–3 cm differs from the rest of the core (all *p* < 0.001) and section 6–7 cm differs from the top part of the core (section 0–1 cm to 5–6 cm, all *p* < 0.05). Overall, the variability in MP size with no significant differences between the oldest and most recent MPs sequestered in the sediment column suggests that MPs have not been subjected to physical degradation. Furthermore, the general decrease in MP occurrence with sediment depth and constant sediment accumulation in steady-state conditions showed by the ^210^Pb profile rules out vertical remobilization of the MPs along the investigated sediment core. In addition, our results on the CI indicate the preservation of MPs, most likely due to a slowdown of weathering effects, particularly photooxidation and hydrolysis. Lastly, these observations support the feasibility of MPs as long-lasting chronostratigraphic markers, as previously suggested in the literature.^18,69,70^ Limitations should be considered. The presence of MPs in sediment records can be used to corroborate geochronologies when the archive shows an undisturbed nature. Additionally, the reliable characterization of MPs requires targeted analytical methods and prevention of cross-contamination that otherwise might mislead the interpretation of the historical records.

## 4 Implications

In considering the limitations of this study, we acknowledge that coastal benthic environments are complex and highly dynamic, with exposure to disturbing natural and anthropogenic events that can alter the MP accumulation in these systems. The main objectives of this study were to investigate the sequestration and long-term fate of small MPs (11–1000 μm) buried in marine sediments. To comply with these research questions, selecting an undisturbed sediment core with a high SR from a relatively high MP-polluted area was indispensable to provide high-resolution data on the fate of buried MPs on a sub-decadal scale. The application of the state-of-the-art FPA-μFTIR-Imaging method for MP characterization and CI computation described in this study provided robust results that indicated that (i) the MP mass sequestered in marine sediments increased exponentially from 1965 to 2019 and (ii) MP properties do not vary over time, suggesting preservation of these pollutants within the sedimentary record.

## Supporting Information Available

Polymer and size class of the microplastic recorded in the blanks; microplastic concentration and blank correction; microplastic extraction protocol; ^210^Pb_xs_ specific activity profile; and Ebro river discharge records from 1951 to 2016 (PDF)

## References

[ref1] Crutzen P. J. The “Anthropocene”. Earth System Science in the Anthropocene; Springer: Berlin, Heidelberg, 2006, 13 −18.

[ref2] Elhacham E.; Ben-Uri L.; Grozovski J.; Bar-On Y. M.; Milo R. Global Human-Made Mass Exceeds All Living Biomass. Nature 2020, 588, 442 10.1038/s41586-020-3010-5. 33299177

[ref3] Persson L.; Carney Almroth B. M.; Collins C. D.; Cornell S.; de Wit C. A.; Diamond M. L.; Fantke P.; Hassellöv M.; MacLeod M.; Ryberg M. W.; Søgaard Jørgensen P.; Villarrubia-Gómez P.; Wang Z.; Hauschild M. Z. Outside the Safe Operating Space of the Planetary Boundary for Novel Entities. Environ. Sci. Technol. 2022, 56, 1510–1521. 10.1021/acs.est.1c04158. 35038861 PMC8811958

[ref4] Zalasiewicz J.; Waters C. N.; Ivar do Sul J. A.; Corcoran P. L.; Barnosky A. D.; Cearreta A.; Edgeworth M.; Gałuszka A.; Jeandel C.; Leinfelder R.; McNeill J. R.; Steffen W.; Summerhayes C.; Wagreich M.; Williams M.; Wolfe A. P.; Yonan Y. The Geological Cycle of Plastics and Their Use as a Stratigraphic Indicator of the Anthropocene. Anthropocene 2016, 13, 4–17. 10.1016/J.ANCENE.2016.01.002.

[ref5] Smeaton C. Augmentation of Global Marine Sedimentary Carbon Storage in the Age of Plastic. Limnol. Oceanogr. Lett. 2021, 6, 113–118. 10.1002/LOL2.10187.

[ref6] Hamm T.; Lenz M. Negative Impacts of Realistic Doses of Spherical and Irregular Microplastics Emerged Late during a 42 Weeks-Long Exposure Experiment with Blue Mussels. Sci. Total Environ. 2021, 778, 146088 10.1016/J.SCITOTENV.2021.146088. 34030367

[ref7] Frias J. P. G. L.; Nash R. Microplastics: Finding a Consensus on the Definition. Mar Pollut Bull. 2019, 138, 145–147. 10.1016/j.marpolbul.2018.11.022. 30660255

[ref8] Zarfl C. Promising Techniques and Open Challenges for Microplastic Identification and Quantification in Environmental Matrices. Anal. Bioanal. Chem. 2019, 411, 3743–3756. 10.1007/s00216-019-01763-9. 30919016

[ref9] Primpke S.; Christiansen S. H.; Cowger W.; de Frond H.; Deshpande A.; Fischer M.; Holland E. B.; Meyns M.; O’Donnell B. A.; Ossmann B. E.; Pittroff M.; Sarau G.; Scholz-Böttcher B. M.; Wiggin K. J. Critical Assessment of Analytical Methods for the Harmonized and Cost-Efficient Analysis of Microplastics. Appl. Spectrosc. 2020, 74, 1012–1047. 10.1177/0003702820921465. 32249594

[ref10] Woodall L. C.; Sanchez-Vidal A.; Canals M.; Paterson G. L. J.; Coppock R.; Sleight V.; Calafat A.; Rogers A. D.; Narayanaswamy B. E.; Thompson R. C. The Deep Sea Is a Major Sink for Microplastic Debris. R. Soc. Open Sci. 2014, 1, 140317 10.1098/rsos.140317. 26064573 PMC4448771

[ref11] Martin C.; Young C. A.; Valluzzi L.; Duarte C. M. Ocean Sediments as the Global Sink for Marine Micro- and Mesoplastics. Limnol. Oceanogr. Lett. 2022, 7, 235–243. 10.1002/LOL2.10257.

[ref12] Pabortsava K.; Lampitt R. S. High Concentrations of Plastic Hidden beneath the Surface of the Atlantic Ocean. Nat. Commun. 2020, 11, 4073 10.1038/s41467-020-17932-9. 32811835 PMC7434887

[ref13] Kane I. A.; Clare M. A. Dispersion, Accumulation, and the Ultimate Fate of Microplastics in Deep-Marine Environments: A Review and Future Directions. Front. Earth Sci. 2019, 7, 80 10.3389/feart.2019.00080.

[ref14] Van Sebille E.; Aliani S.; Law K. L.; Maximenko N.; Alsina J. M.; Bagaev A.; Bergmann M.; Chapron B.; Chubarenko I.; Cózar A.; Delandmeter P.; Egger M.; Fox-Kemper B.; Garaba S. P.; Goddijn-Murphy L.; Hardesty B. D.; Hoffman M. J.; Isobe A.; Jongedijk C. E.; Kaandorp M. L. A.; Khatmullina L.; Koelmans A. A.; Kukulka T.; Laufkötter C.; Lebreton L.; Lobelle D.; Maes C.; Martinez-Vicente V.; Morales Maqueda M. A.; Poulain-Zarcos M.; Rodríguez E.; Ryan P. G.; Shanks A. L.; Shim W. J.; Suaria G.; Thiel M.; van den Bremer T. S.; Wichmann D. The Physical Oceanography of the Transport of Floating Marine Debris. Environ. Res. Lett. 2020, 15, 023003 10.1088/1748-9326/ab6d7d.

[ref15] Martin J.; Lusher A. L.; Nixon F. C. A Review of the Use of Microplastics in Reconstructing Dated Sedimentary Archives. Sci. Total Environ. 2021, 806, 150818 10.1016/J.SCITOTENV.2021.150818. 34637878

[ref16] Cau A.; Avio C. G.; Dessì C.; Follesa M. C.; Moccia D.; Regoli F.; Pusceddu A. Microplastics in the Crustaceans Nephrops Norvegicus and Aristeus Antennatus: Flagship Species for Deep-Sea Environments?. Environ. Pollut. 2019, 255, 113107 10.1016/j.envpol.2019.113107. 31671310

[ref17] Carreras-Colom E.; Constenla M.; Soler-Membrives A.; Cartes J. E.; Baeza M.; Carrass On M. A Closer Look at Anthropogenic Fiber Ingestion in *Aristeus Antennatus* in the NW Mediterranean Sea: Differences among Years and Locations and Impact on Health Condition *. Environ. Pollut. 2020, 263, 114567 10.1016/j.envpol.2020.114567. 33618489

[ref18] Bancone C. E. P.; Turner S. D.; Ivar do Sul J. A.; Rose N. L. The Paleoecology of Microplastic Contamination. Front. Environ. Sci. 2020, 8, 574008 10.3389/fenvs.2020.574008.

[ref19] Simon-Sánchez L.; Grelaud M.; Garcia-Orellana J.; Ziveri P. River Deltas as Hotspots of Microplastic Accumulation: The Case Study of the Ebro River (NW Mediterranean). Sci. Total Environ. 2019, 687, 1186–1196. 10.1016/j.scitotenv.2019.06.168. 31412454

[ref20] Löder M. G. J.; Kuczera M.; Mintenig S.; Lorenz C.; Gerdts G. Focal Plane Array Detector-Based Micro-Fourier-Transform Infrared Imaging for the Analysis of Microplastics in Environmental Samples. Environ. Chem. 2015, 12, 563–581. 10.1071/EN14205.

[ref21] Geyer R.; Jambeck J. R.; Law K. L. Production, Use, and Fate of All Plastics Ever Made. Sci. Adv. 2017, 3, e1700782 10.1126/sciadv.1700782. 28776036 PMC5517107

[ref22] Abel S. M.; Primpke S.; Int-Veen I.; Brandt A.; Gerdts G. Systematic Identification of Microplastics in Abyssal and Hadal Sediments of the Kuril Kamchatka Trench. Environ. Pollut. 2021, 269, 116095 10.1016/j.envpol.2020.116095. 33257152

[ref23] Int-Veen I.; Nogueira P.; Isigkeit J.; Hanel R.; Kammann U. Positively Buoyant but Sinking: Polymer Identification and Composition of Marine Litter at the Seafloor of the North Sea and Baltic Sea. Mar. Pollut. Bull. 2021, 172, 112876 10.1016/J.MARPOLBUL.2021.112876. 34450407

[ref24] Sanchez-Cabeza J. A.; Masqué P.; Ani-Ragolta I. 210Pb And210Po Analysis in Sediments and Soils by Microwave Acid Digestion. J. Radioanal. Nucl. Chem. 1998, 227, 19–22. 10.1007/BF02386425.

[ref25] Krishnaswamy S.; Lal D.; Martin J. M.; Meybeck M. Geochronology of Lake Sediments. Earth Planet. Sci. Lett. 1971, 11, 407–414. 10.1016/0012-821X(71)90202-0.

[ref26] Liu F.; Vianello A.; Vollertsen J. Retention of Microplastics in Sediments of Urban and Highway Stormwater Retention Ponds. Environ. Pollut. 2019, 255, 113335 10.1016/j.envpol.2019.113335. 31604201

[ref27] Primpke S.; Cross R. K.; Mintenig S. M.; Simon M.; Vianello A.; Gerdts G.; Vollertsen J. Toward the Systematic Identification of Microplastics in the Environment: Evaluation of a New Independent Software Tool (SiMPle) for Spectroscopic Analysis. Appl. Spectrosc. 2020, 74, 1127–1138. 10.1177/0003702820917760. 32193948 PMC7604885

[ref28] Primpke S.; Wirth M.; Lorenz C.; Gerdts G. Reference Database Design for the Automated Analysis of Microplastic Samples Based on Fourier Transform Infrared (FTIR) Spectroscopy. Anal. Bioanal. Chem. 2018, 410, 5131–5141. 10.1007/s00216-018-1156-x. 29978249 PMC6113679

[ref29] Liu F.; Olesen K. B.; Borregaard A. R.; Vollertsen J. Microplastics in Urban and Highway Stormwater Retention Ponds. Sci. Total Environ. 2019, 671, 992–1000. 10.1016/j.scitotenv.2019.03.416. 31604201

[ref30] Simon M.; van Alst N.; Vollertsen J. Quantification of Microplastic Mass and Removal Rates at Wastewater Treatment Plants Applying Focal Plane Array (FPA)-Based Fourier Transform Infrared (FT-IR) Imaging. Water Res. 2018, 142, 1–9. 10.1016/J.WATRES.2018.05.019. 29804032

[ref31] Vianello A.; Jensen R. L.; Liu L.; Vollertsen J. Simulating Human Exposure to Indoor Airborne Microplastics Using a Breathing Thermal Manikin. Sci. Rep. 2019, 9, 8670 10.1038/s41598-019-45054-w. 31209244 PMC6573036

[ref32] Lorenz C.; Roscher L.; Meyer M. S.; Hildebrandt L.; Prume J.; Löder M. G. J.; Primpke S.; Gerdts G. Spatial Distribution of Microplastics in Sediments and Surface Waters of the Southern North Sea. Environ. Pollut. 2019, 252, 1719–1729. 10.1016/j.envpol.2019.06.093. 31284214

[ref33] QGIS Development Team. QGIS Geographic Information System. Open Source Geospatial Foundation Project 2020.

[ref34] RStudio Team. RStudio: Integrated Development for R. RStudio. RStudio: Integrated Development for R. RStudio; PBC: Boston, MA 2020.

[ref35] Wickham H. Ggplot2: Elegant Graphics for Data Analysis; Springer-Verlag: New York 2016.

[ref36] Wilke C. Cowplot: Streamlined Plot Theme and Plot Annotations for “Ggplot2”. 2020.

[ref37] Andrady A. L. Microplastics in the Marine Environment. Mar. Pollut. Bull. 2011, 62, 1596–1605. 10.1016/j.marpolbul.2011.05.030. 21742351

[ref38] ter Halle A.; Ladirat L.; Martignac M.; Mingotaud A. F.; Boyron O.; Perez E. To What Extent Are Microplastics from the Open Ocean Weathered?. Environ. Pollut. 2017, 227, 167–174. 10.1016/j.envpol.2017.04.051. 28460234

[ref39] Almond J.; Sugumaar P.; Wenzel M. N.; Hill G.; Wallis C. Determination of the Carbonyl Index of Polyethylene and Polypropylene Using Specified Area under Band Methodology with ATR-FTIR Spectroscopy. E-Polymers 2020, 20, 369–381. 10.1515/epoly-2020-0041.

[ref40] Menges F. Spectragryph-Optical Spectroscopy Software Version, 1(5). 2019.

[ref41] Arias-Ortiz A.; Masqué P.; Garcia-Orellana J.; Serrano O.; Mazarrasa I.; Marbá N.; Lovelock C. E.; Lavery P. S.; Duarte C. M. Reviews and Syntheses: 210Pb-Derived Sediment and Carbon Accumulation Rates in Vegetated Coastal Ecosystems - Setting the Record Straight. Biogeosciences 2018, 15, 6791–6818. 10.5194/BG-15-6791-2018.

[ref42] Zografos C. Flows of Sediment, Flows of Insecurity: Climate Change Adaptation and the Social Contract in the Ebro Delta, Catalonia. Geoforum 2017, 80, 49–60. 10.1016/j.geoforum.2017.01.004.

[ref43] Bergmann M.; Wirzberger V.; Krumpen T.; Lorenz C.; Primpke S.; Tekman M. B.; Gerdts G. High Quantities of Microplastic in Arctic Deep-Sea Sediments from the HAUSGARTEN Observatory. Environ. Sci. Technol. 2017, 51, 11000–11010. 10.1021/acs.est.7b03331. 28816440

[ref44] Vianello A.; Boldrin A.; Guerriero P.; Moschino V.; Rella R.; Sturaro A.; da Ros L. Microplastic Particles in Sediments of Lagoon of Venice, Italy: First Observations on Occurrence, Spatial Patterns and Identification. Estuar. Coast. Shelf Sci. 2013, 130, 54–61. 10.1016/j.ecss.2013.03.022.

[ref45] Kaandorp M. L. A.; Dijkstra H. A.; van Sebille E. Closing the Mediterranean Marine Floating Plastic Mass Budget: Inverse Modeling of Sources and Sinks. Environ. Sci. Technol. 2020, 54, 11980–11989. 10.1021/acs.est.0c01984. 32852202 PMC7547878

[ref46] Foufoula-Georgiou E.; Syvitski J.; Paola C.; Hoanh C. T.; Tuong P.; Vörösmarty C.; Kremer H.; Brondizio E.; Saito Y.; Twilley R. International Year of Deltas 2013: A Proposal. Eos, Trans. Am. Geophys. Union 2011, 92, 340–341. 10.1029/2011EO400006.

[ref47] Brandon J. A.; Jones W.; Ohman M. D. Multidecadal Increase in Plastic Particles in Coastal Ocean Sediments. Sci. Adv. 2019, 5, eaax0587 10.1126/sciadv.aax0587. 31517049 PMC6726453

[ref48] Dahl M.; Bergman S.; Björk M.; Diaz-Almela E.; Granberg M.; Gullström M.; Leiva-Dueñas C.; Magnusson K.; Marco-Méndez C.; Piñeiro-Juncal N.; Mateo M. Á. A Temporal Record of Microplastic Pollution in Mediterranean Seagrass Soils. Environ. Pollut. 2021, 273, 116451 10.1016/J.ENVPOL.2021.116451. 33486243

[ref49] van der Hal N.; Ariel A.; Angel D. L. Exceptionally High Abundances of Microplastics in the Oligotrophic Israeli Mediterranean Coastal Waters. Mar. Pollut. Bull. 2017, 116, 151–155. 10.1016/j.marpolbul.2016.12.052. 28063700

[ref50] Korez Š.; Gutow L.; Saborowski R. Microplastics at the Strandlines of Slovenian Beaches. Mar. Pollut. Bull. 2019, 145, 334–342. 10.1016/j.marpolbul.2019.05.054. 31590795

[ref51] Vianello A.; Da Ros L.; Boldrin A.; Marceta T.; Moschino V. First Evaluation of Floating Microplastics in the Northwestern Adriatic Sea. Environ. Sci. Pollut. Res. 2018, 25, 28546–28561. 10.1007/s11356-018-2812-6. 30091074

[ref52] Hartmann N. B.; Hüffer T.; Thompson R. C.; Hassellöv M.; Verschoor A.; Daugaard A. E.; Rist S.; Karlsson T.; Brennholt N.; Cole M.; Herrling M. P.; Hess M. C.; Ivleva N. P.; Lusher A. L.; Wagner M. Are We Speaking the Same Language? Recommendations for a Definition and Categorization Framework for Plastic Debris. Environ. Sci. Technol. 2019, 53, 1039–1047. 10.1021/acs.est.8b05297. 30608663

[ref53] Rochman C. M.; Brookson C.; Bikker J.; Djuric N.; Earn A.; Bucci K.; Athey S.; Huntington A.; McIlwraith H.; Munno K.; De Frond H.; Kolomijeca A.; Erdle L.; Grbic J.; Bayoumi M.; Borrelle S. B.; Wu T.; Santoro S.; Werbowski L. M.; Zhu X.; Giles R. K.; Hamilton B. M.; Thaysen C.; Kaura A.; Klasios N.; Ead L.; Kim J.; Sherlock C.; Ho A.; Hung C. Rethinking Microplastics as a Diverse Contaminant Suite. Environ. Toxicol. Chem. 2019, 38, 703–711. 10.1002/etc.4371. 30909321

[ref54] Simon-Sánchez L.; Grelaud M.; Franci M.; Ziveri P. Are Research Methods Shaping Our Understanding of Microplastic Pollution? A Literature Review on the Seawater and Sediment Bodies of the Mediterranean Sea. Environ. Pollut. 2022, 292, 118275 10.1016/j.envpol.2021.118275. 34626717

[ref55] Martin C.; Baalkhuyur F.; Valluzzi L.; Saderne V.; Cusack M.; Almahasheer H.; Krishnakumar P. K.; Rabaoui L.; Qurban M. A.; Arias-Ortiz A.; Masqué P.; Duarte C. M. Exponential Increase of Plastic Burial in Mangrove Sediments as a Major Plastic Sink. Sci. Adv. 2020, 6, eaaz5593 10.1126/SCIADV.AAZ5593. 33115749 PMC7608790

[ref56] Egger M.; Nijhof R.; Quiros L.; Leone G.; Royer S.-J.; McWhirter A. C.; Kantakov G. A.; Radchenko V. I.; Pakhomov E. A.; Hunt B. P. V.; Lebreton L. A Spatially Variable Scarcity of Floating Microplastics in the Eastern North Pacific Ocean. Environ. Res. Lett. 2020, 15, 114056 10.1088/1748-9326/abbb4f.

[ref57] Cózar A.; Echevarría F.; González-Gordillo J. I.; Irigoien X.; Úbeda B.; Hernández-León S.; Palma Á. T.; Navarro S.; García-de-Lomas J.; Ruiz A.; Fernández-de-Puelles M. L.; Duarte C. M. Plastic Debris in the Open Ocean. Proc. Natl. Acad. Sci. U. S. A. 2014, 111, 10239–10244. 10.1073/pnas.1314705111. 24982135 PMC4104848

[ref58] Egger M.; Schilt B.; Wolter H.; Mani T.; de Vries R.; Zettler E.; Niemann H. Pelagic Distribution of Plastic Debris (> 500 Mm) and Marine Organisms in the Upper Layer of the North Atlantic Ocean. Sci. Rep. 2022, 12, 13465 10.1038/s41598-022-17742-7. 35953623 PMC9372048

[ref59] Kooi M.; Reisser J.; Slat B.; Ferrari F. F.; Schmid M. S.; Cunsolo S.; Brambini R.; Noble K.; Sirks L. A.; Linders T. E. W.; Schoeneich-Argent R. I.; Koelmans A. A. The Effect of Particle Properties on the Depth Profile of Buoyant Plastics in the Ocean. Sci. Rep. 2016, 6, 33882 10.1038/srep33882. 27721460 PMC5056413

[ref60] Reisser J.; Slat B.; Noble K.; Du Plessis K.; Epp M.; Proietti M.; De Sonneville J.; Becker T.; Pattiaratchi C. The Vertical Distribution of Buoyant Plastics at Sea: An Observational Study in the North Atlantic Gyre. Biogeosciences 2015, 12, 1249–1256. 10.5194/bg-12-1249-2015.

[ref61] Kukkola A. T.; Senior G.; Maes T.; Silburn B.; Bakir A.; Kröger S.; Mayes A. G. A Large-Scale Study of Microplastic Abundance in Sediment Cores from the UK Continental Shelf and Slope. Mar. Pollut. Bull. 2022, 178, 113554 10.1016/J.MARPOLBUL.2022.113554. 35390630

[ref62] Dong M.; Luo Z.; Jiang Q.; Xing X.; Zhang Q.; Sun Y. The Rapid Increases in Microplastics in Urban Lake Sediments. Sci. Rep. 2020, 10, 848 10.1038/s41598-020-57933-8. 31964973 PMC6972887

[ref63] Courtene-Jones W.; Quinn B.; Ewins C.; Gary S. F.; Narayanaswamy B. E. Microplastic Accumulation in Deep-Sea Sediments from the Rockall Trough. Mar. Pollut. Bull. 2020, 154, 111092 10.1016/j.marpolbul.2020.111092. 32319921

[ref64] Song Y. K.; Hong S. H.; Jang M.; Han G. M.; Rani M.; Lee J.; Shim W. J. A Comparison of Microscopic and Spectroscopic Identification Methods for Analysis of Microplastics in Environmental Samples. Mar. Pollut. Bull. 2015, 93, 202–209. 10.1016/j.marpolbul.2015.01.015. 25682567

[ref65] Torre M.; Digka N.; Anastasopoulou A.; Tsangaris C.; Mytilineou C. Anthropogenic Microfibres Pollution in Marine Biota. A New and Simple Methodology to Minimize Airborne Contamination. Mar. Pollut. Bull. 2016, 113, 55–61. 10.1016/J.MARPOLBUL.2016.07.050. 27491365

[ref66] Brandon J.; Goldstein M.; Ohman M. D. Long-Term Aging and Degradation of Microplastic Particles: Comparing in Situ Oceanic and Experimental Weathering Patterns. Mar. Pollut. Bull. 2016, 110, 299–308. 10.1016/j.marpolbul.2016.06.048. 27344287

[ref67] Kim S.; Sin A.; Nam H.; Park Y.; Lee H.; Han C. Advanced Oxidation Processes for Microplastics Degradation: A Recent Trend. Chem. Eng. J. Adv. 2022, 9, 100213 10.1016/J.CEJA.2021.100213.

[ref68] Potrykus M.; Redko V.; Głowacka K.; Piotrowicz-Cieślak A.; Szarlej P.; Janik H.; Wolska L. Polypropylene Structure Alterations after 5 Years of Natural Degradation in a Waste Landfill. Sci. Total Environ. 2021, 758, 143649 10.1016/j.scitotenv.2020.143649. 33293087

[ref69] Ivar do Sul J. A.; Labrenz M. Microplastics into the Anthropocene. Handbook of Microplastics in the Environment; Springer International Publishing, 2021, 1 −16.

[ref70] Corcoran P. L.; Norris T.; Ceccanese T.; Walzak M. J.; Helm P. A.; Marvin C. H. Hidden Plastics of Lake Ontario, Canada and Their Potential Preservation in the Sediment Record. Environ. Pollut. 2015, 204, 17–25. 10.1016/J.ENVPOL.2015.04.009. 25898233

