# Supplementary material for: Can a Sediment Core Reveal the Plastic Age? Microplastic Preservation in a Coastal Sedimentary Record
Source: Environ Sci Technol. 2022 Nov 14;56(23):16780–8. doi: 10.1021/acs.est.2c04264 (PMC9730841; doi:10.1021/acs.est.2c04264)
Supplement: Supplementary file 1 — es2c04264_si_001.pdf [file es2c04264_si_001.pdf]

Can a sediment core reveal the Plastic Age? – Microplastic preservation in a coastal sedimentary record

Laura Simon-Sánchez* [1], Michaël Grelaud [1], Claudia Lorenz [2], Jordi Garcia-Orellana[1,3], Alvise Vianello[2], Fan Liu [2], Jes Vollertsen [2], Patrizia Ziveri[1, 4]

[1]Institute of Environmental Science and Technology (ICTA), Autonomous University of Barcelona (UAB), Bellaterra 08193, Spain
[2]Aalborg University, Department of the Built Environment, Thomas Manns Vej 23, 9220 Aalborg Øst, Denmark
[3]Departament de Física, Universitat Autònoma de Barcelona, Autonomous University of Barcelona (UAB), Bellaterra 08193, Spain
[4]Catalan Institution for Research and Advanced Studies (ICREA), Pg. Lluís Companys 23, Barcelona 08010, Spain

Corresponding author: laura.simon@uab.cat

Supplementary material

Table S1. Polymer and size class of the microplastic recorded in the blanks 2

Table S2. Microplastic concentration and blank correction. ss. 3

Supplementary text. Microplastic extraction protocol...3

Figure S1. $^{210}Pb_{xs}$ specific activity profile of the sediment core MERS_BI_ST17_MUC2...4

Figure S2. Ebro river discharge records from 1951 to 2016 were recorded at the stream gauge of Tortosa...5

Table S1. Polymer and size class of the microplastic recorded in the blanks

Sample	Polymer	Size class (um)																				Total
		11-25	25-50	50-75	75-100	100-125	125-150	150-175	175-200	200-225	225-250	250-275	275-300	325-350	350-375	375-400	400-425	425-450	450-475	475-500	500-1000	
PB01	Polyester	0	1	1	1	1	0	0	0	0	0	0	0	0	0	0	0	0	0	0	1	5
	PS	0	0	1	0	0	0	0	0	0	0	0	0	0	0	0	0	0	0	0	0	1
PB02	Polyester	0	2	0	2	0	1	0	0	0	0	0	1	0	0	0	0	0	0	0	1	7
	PP	1	1	3	1	0	0	0	0	0	0	0	0	0	0	0	0	0	0	0	0	6
Pre-Plastic (34-35 cm)	Polyester	0	0	1	0	1	1	1	0	0	0	0	0	0	0	0	0	0	0	0	0	4
	PE	0	0	0	0	0	0	0	0	0	0	0	0	0	0	1	0	0	0	0	0	1
	PP	0	0	1	1	0	0	0	0	0	0	0	0	0	0	0	0	0	0	0	0	2

Table S2. Microplastic concentration and blank correction. The table presents the data on the number of particles and mass found on the blanks and the samples. The blank correction was applied considering the MPs found in the procedural blank run along with the batch of samples and the MPs found in section 34-35 cm representing the potential contamination during the sampling. Note that the absolute number of the blanks does not always correspond to the number of particles subtracted because the blank correction was applied per polymer and size class.

	ID	Dry mass (g)	Correction applied	MPs No Corrected (n)	MPs corrected (n)	MP mass No corrected (ng)	MP Mass corrected (ng)
Blanks	PB02	NA	NA	13	13	2741.98737	2741.98737
	PB01	NA	NA	6	6	1231.76903	1231.76903
	Pre-Plastics 34-35cm		PB02	7	4	5283.22834	5104.23059
Samples	0-1 cm	22.768	PB02+34-35 cm	174	158	13963.2174	12872.0385
	1-2 cm	35.03	PB02+34-35cm	74	61	116256.838	50164.7075
	2-3 cm	44.31	PB01+34-35cm	37	32	377312.365	33209.2504
	3-4 cm	45.849	PB02+34-35cm	226	209	26620.1472	17223.6729
	5-6 cm	52.346	PB02+34-35cm	154	141	13038.9305	12502.0483
	6-7 cm	58.565	PB01+34-35cm	225	217	10944.117	7413.83108
	7-8 cm	59.508	PB02+34-35cm	51	42	4404.10945	2978.99285
	8-9 cm	58.925	PB01+34-35cm	46	42	2991.06673	2938.55209

Supplementary text. Microplastic extraction protocol

The sediment mass processed for MP analysis varied between slices, ranging between 22.8 and 59.5 g. The samples were placed in 2 L beakers and pre-oxidized using a peroxide-based treatment (10%H_2O_2). The pre-oxidation step was continued over a week or until no foaming was observed while adding H_2O_2. Then, the samples were wet sieved using a 1 mm sieve. Due to the large volume of milli-Q water used for the sieving, the samples were left to settle for over a week. The supernatant was filtered into a 10 µm stainless steel filter, and the collected particles were transferred with the settled solids (<1 mm) into a tray for oven-drying (50° C) for five days.

Once the samples were dried, they underwent a multi-step protocol based on density separation ($ZnCl_2$), multi-enzymatic buffered treatments (Protease, Cellulose, and Viscozyme), and catalyzed oxidation. Every sample was resuspended in $ZnCl_2$ via sonication and transferred to a 2 L glass separatory funnel. The sample was aerated for 30 min using pre-filtered air fluxed from the bottom of the funnel and then left to settle overnight. The settled material was discharged from the funnel using the stopcock, and the supernatant containing the floating solids was then collected into a 2 L beaker. This step was repeated twice to enhance the recovery of the particles. The aliquots of supernatant collected in both repetitions were combined and filtered onto the 10 µm stainless steel filter used in the previous step. The filter was sonicated in 300 mL of Sodium Dodecyl Sulfate solution (SDS, 5% w/vol) to resuspend the particles and incubated for at least 40 hours at 50º C in a water bath. Subsequently, the sample was filtered onto the filter, and the retained material was resuspended in TRIS buffer solution (pH 8.2) and incubated (50° C; min. 40 hours) after adding 0.5 mL of Protease (Protease from Bacillus sp.®, Sigma-Aldrich). The

sample was then filtered and the filter resuspended in acetate buffer (pH 4.8) and re-incubated after adding 0.5 mL Cellulase blend (Cellulase enzyme blend®, Sigma-Aldrich) and 0.5mL of Viscozyme (Viscozyme®L, Sigma-Aldrich). After a minimum of 40 hours of incubation, the samples were filtered and resuspended in 200 mL of pre-filtered milli-Q water and submitted to Fenton reaction (145 mL 50% H_2O_2 + 65 mL of 0.1M NaOH, + 62 mL of 0.1M $FeSO_4$). The reaction was closely monitored to maintain room temperature (20-30° C) using an ice-water bath. After 24 hours, the samples were filtered as previously described, and the particles were resuspended in $ZnCl_2$ for a second-density separation using 250 mL separatory funnels. The settled solids were discarded, and the supernatant was collected for filtering, similar to the previous steps. After resuspending the particles in a small volume of 50% of HPLC ethanol, the enriched liquid was transferred in aliquots into a glass 10 mL headspace vial. The solvent was evaporated using an evaporator (TurboVap® LV, Biotage). Finally, a fixed volume (3 mL of HPLC grade 50% Ethanol) was used to re-mobilize the particles.

Figure S1. $^{210}Pb_{xs}$ specific activity profile of the sediment core MERS_BI_ST17_MUC2

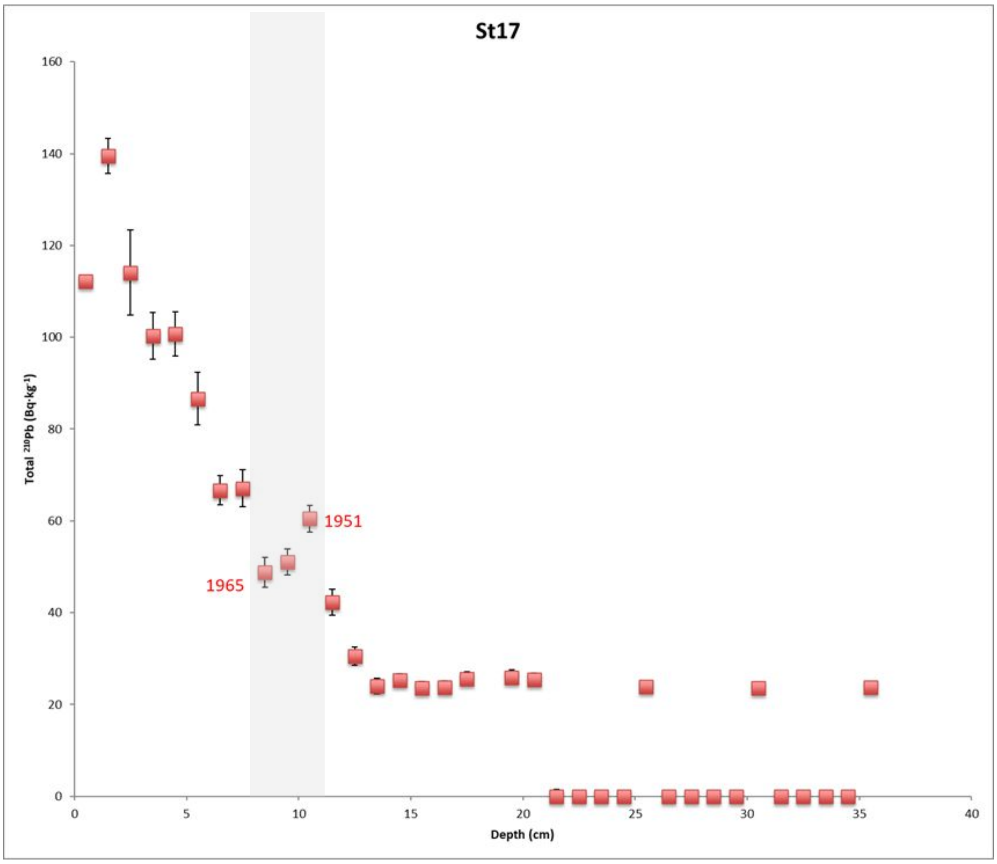

Fig S1. $^{210}Pb_{xs}$ specific activity profile of the sediment core MERS_BI_ST17_MUC2. The grey box highlights the anomaly on the exponential trend characterizing the sediment accumulation in steady-state conditions. In the highlighted area, a change in the sedimentation rate can be observed, most likely due to the construction of the Mequinenza and Ribaroja dams.

Figure S2. Ebro river discharge records from 1951 to 2016 were recorded at the stream gauge of Tortosa

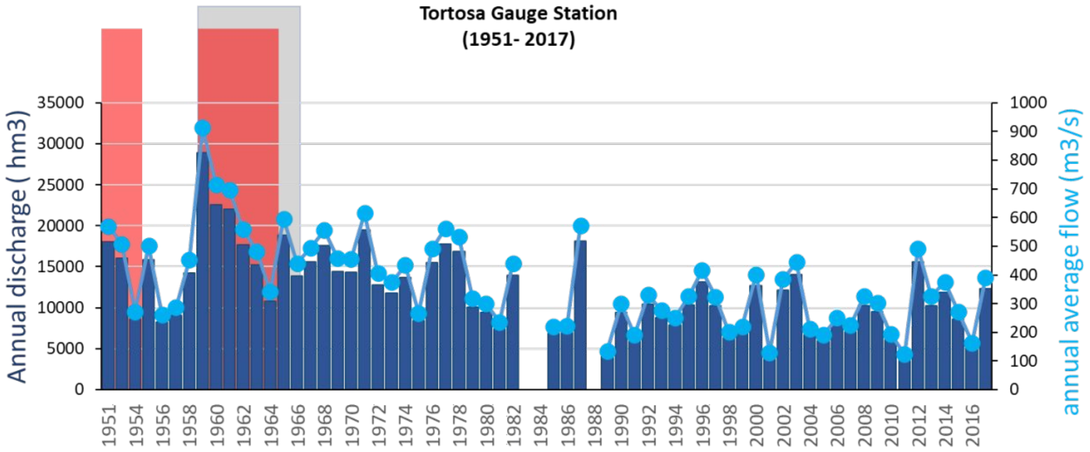

Fig S2. Ebro river discharge records from 1951 to 2016 were recorded at the stream gauge of Tortosa. The red boxes highlight the decrease in the annual flow of the Ebro River, whereas the grey box the period of the dams' construction (1958-1966).
